# Exploring the real-world experience of abemaciclib treatment for HR +, HER2 − metastatic breast cancer—a qualitative analysis of the IMPACTOR study

**DOI:** 10.1007/s00520-025-09444-3

**Published:** 2025-04-26

**Authors:** Rachel Starkings, Helena Harder, Lesley Fallowfield, Valerie Shilling

**Affiliations:** SHORE-C, Brighton and Sussex Medical School, University of Brighton and University of Sussex, Brighton, UK

**Keywords:** Abemaciclib, Coping, Diarrhoea management, Metastatic breast cancer, Qualitative, Side effects

## Abstract

**Purpose:**

The IMPACTOR study (IMPact of AbemaCiclib on patienTs’ rOles and Responsibilities–ISRCTN17281696) was developed to capture experiences of women with MBC being treated with abemaciclib in a real-world setting. The primary aim was to explore changes to quality of life over time and our secondary aim was to understand these changes in detail via qualitative interviews, as presented here.

**Methods:**

A singular interview was offered to participants who had expressed an interest at the point of consent. These were all conducted remotely using a semi-structured interview topic guide.

**Results:**

Twenty interviews were completed and analysed using a framework approach to thematic analysis. Eight themes were developed—COVID-19, experience of MBC, side effects, side effect management, treatment information and support, relationship impacts, impact on daily life, and finances and employment.

**Conclusions:**

It was apparent that participants faced side effects from treatment but undertook steps to manage these as much as possible. Adaptations were often led by a belief about the benefits of remaining on treatment. Adjustments ranged from modifying routines to carrying personal hygiene supplies when out in public in case of diarrhoea. While this was anticipated, other side effects were less well known with variable clinical support and available information. Family support was raised frequently, predominantly in relation to the impact MBC had on roles and relationships. Themes from this work can be thought of via theories about treatment belief and adherence, such as the common-sense and self-regulation models, as participants reflected on both emotional and cognitive coping strategies.

Trial registration - ISRCTN17281696.

**Supplementary Information:**

The online version contains supplementary material available at 10.1007/s00520-025-09444-3.

## Introduction

Effective treatment for metastatic breast cancer (MBC) has significantly advanced in recent years, with more available options offering extended progression free survival (PFS) [1, 2]. However, side effects are common, requiring additional management and support [2, 3]. CDK4/6 inhibitors levy a high symptom burden, yet are the gold standard for HR +, HER2 − MBC treatment, when combined with endocrine therapy (ET) [4–12]. Abemaciclib in particular is associated with gastrointestinal side effects, most notably diarrhoea, potentially explaining the higher rates of discontinuation compared to other CDK4/6 inhibitors [4, 6, 13]. There are discrepancies in the limited published literature about the impact of treatment side effects on patient quality of life, with some suggesting impairment, others none [5, 14].

The majority of knowledge about CDK4/6 inhibitors, and their side effects, comes from trial data and clinical practice, using physician reported grading [15, 16]. Real-world data originates predominantly from the USA and focuses on treatment with palbociclib [10]. There are other studies capturing data about survival and side effects but these do not include information from the patient perspective [17–19]. There is a clear gap then in our understanding of what life is like for patients taking abemaciclib, beyond clinical and quantitative reports of adverse events or adherence.

The IMPACTOR study (IMPact of AbemaCiclib on patienTs’ rOles and Responsibilities–ISRCTN17281696) was developed to capture the impacts of abemaciclib on women with MBC and the ways in which these were managed. The primary aim was to chart changes in quality of life (QoL) over time, using validated questionnaires and diarrhoea diaries. Semi-structured interviews addressed a secondary aim of permitting patients to expand on their positive and negative treatment experiences. Full study details, including primary aims and quantitative assessments, are presented elsewhere [20]; here, the focus is on the qualitative findings stemming from these interviews. This paper follows the SRQR format of reporting qualitative data [21].

## Materials and methods

IMPACTOR was open to recruitment between September 2020 and November 2022. We worked with participant identification centres who would approach eligible patients to gauge their interest in the study. We were alerted to those who wanted to learn more and approached them to discuss the study. Participants were eligible if they had recently been prescribed abemaciclib, either with fulvestrant or an aromatase inhibitor. They were not assigned treatment based on joining this study. Participants needed to be 18 or older, able to consent, and read/speak in English. We also excluded those who were too distressed or were inpatients. Participants were enrolled in the study for 25 weeks and could, at the point of consent, register an interest in an optional singular telephone interview. Participants were not compensated for their part in either the wider study or the interview.

Full details of study assessments, intervals, and participant demographics can be found in the companion paper [20] but interview demographics are presented in Table 1. There were 44 women in the main study, 39 expressed an interest in the interview, and 20 took part. Of the 19 individuals who did not complete an interview, 6 were lost to follow up or did not reply to the invitation, 11 withdrew and it was not possible or appropriate to complete an interview, and 2 were overwhelmed. Of the 20 interviews, the majority (12/60%) were conducted between weeks 13 and 16 of participation, capturing people who had been on treatment for at least 3 months. Our initial aim was to conduct all interviews in this window; however, we were pragmatic in this approach and worked with participants to find times which were convenient for them. As such, a further six (30%) were conducted between weeks 17 and 25. Two participants (10%) took part at the point of their study withdrawal as they discontinued treatment. Interviews took an average of 33 min to complete with a range of 18 to 61 min.

**Table 1:** Interview demographics and clinical characteristics (N = 20)

Sociodemographic characteristics	
**Age in years** Mean (SD), median, range	58.85 (9.3), 59, 42–81
**Relationship status, *n* (%)**	
With partner No partner	13 (65) 7 (35)
**Employment status, *n* (%)**	
Full-time work Part-time work Self-employed Long-term sick leave Not-in-paid employment Retired	3 (15) 3 (15) 1 (5) 6 (30) 1 (5) 6 (30)
**Clinical characteristics**	
**Time since diagnosis, *n* (%)**	
< year > 2 years	7 (35) 13 (65)
**Treatment history, *n* (%)**	
Surgery Chemotherapy Radiotherapy	13 (65) 15 (75) 10 (50)
**Other health condition**	10 (50)
**Other medication**	7 (35)

Interviewers (RS, HH, VS) followed a semi-structured approach, using a topic guide to provide some prompts for participants but also following the interviewee in the topics they felt were important to convey. This guide was developed based on the literature available, previous guides we developed for the metastatic setting and with input from three advisors with lived experience contributing to the project. Interviews began by asking participants about their experiences of treatment. The researchers then could explore what the participant had said or introduce external questions if the participant was struggling to know what to say. These additional prompts were centred on different domains such as side effects, impact of treatment on everyday roles, impact to family life, and finances. All participants were asked at the end of the interview if there was anything they wanted to mention or felt we had not covered. An example of the topic guide has been provided as supplementary material (S1).

Interviews were recorded on digital dictaphones before being transcribed. Audio recordings and transcripts, prior to being anonymised, were stored under encryption. Two researchers (RS/HH) listened to these throughout the interviewing process to understand whether any adjustments were needed to the topic guide and/or if there was a saturation point. By reviewing the interviews throughout the process, researchers were able to ensure internal consistency with their approaches and application of the topic guide. Once transcripts were confirmed as accurate, and identifiable details were removed, audio files were destroyed.

Transcripts were analysed using a framework approach to thematic analysis [22]. In line with the aim of this real-world research, we felt a thematic analysis mirrored the constructivist underpinnings of the work [23]. Our goal was to explore how people viewed the treatment, its side effects, and the strategies they implemented to cope. This was an open-ended exploration, rather than a search to determine which strategies were more or less worthwhile.

The first two stages of framework analysis are transcription and immersion in the transcripts, which we completed throughout the interviews, noted above. To complete stage 3, two researchers (RS/HH) double coded 10% of the interviews in the first instance, beginning codebook development. Stage 4 is the development of a framework and the researchers met to discuss what codes were developed, assessing these for overlap or divergence. We agreed a codebook to take forward; a further two interviews were double coded, reviewed, and codebook refinements were made. These changes were guided by Kappa; any nodes that had greater than 2% disagreement or a Kappa lower than 0.75 were discussed. Stage 5 is the application of the framework, done by RS who completed the remainder of coding. Stage 6 is the charting of data; following completion of the coding, RS produced a framework matrix [24]. All three researchers (RS/HH/VS) met to review this alongside the codebook. This provided an opportunity to ensure consensus and resolve any disagreements. Code summaries were produced by RS and reviewed by HH/VS for agreement. The final summaries were then reviewed again with the group convening to discuss and resolve any disagreements. Based on the summaries, RS developed an initial thematic structure and all three researchers met to agree this, making changes together. The final structure comprised 8 main themes and 23 sub-themes (supplementary material). This completed the final stage of analysis or the interpretation of the data.

### Reflexivity statement

The three interviewers (RS, HH, VS) are all experienced in conducting these types of conversations in the research space. None of the researchers had medical training, which was advantageous in limiting bias in the research. Participants were informed that the researchers were not members of their clinical team. This allowed RS, VS, and HH to adopt a position of neutrality when listening to patient stories. It is important to acknowledge the interpersonal reflexivity in this scenario. There are power dynamics inherent in this work and demand characteristics which arise from this. By using a semi-structured topic guide which began with a broad topic, the researchers were positioned as open and curious about the participant’s experience. RS, VS, and HH acknowledged their awareness that diarrhoea was a prominent side effect associated with abemaciclib so the topic guide was also structured to avoid sole focus on this area. Instead participants were prompted to talk about side effects broadly and the researchers would then follow their priorities.

## Results

The eight themes developed from the data were COVID- 19, experience of MBC, side effects, side effect management, treatment information and support, relationship impacts, impact on daily life, and finances and employment. Details of these are provided here with supporting quotes referenced (Tables 2, 3, 4, and 5).

**Table 2:** Supporting quotes for themes of COVID and experience of MBC

Theme	Sub-theme	Ref	Illustrative quote
**COVID**	**N/A**	Q1	But I don’t know whether it’s because of COVID and you can’t talk to your consultant face to face and you don’t see them. It’s hard when you’re doing it on a two-minute telephone call. ID 19, Week 13, 62 years old (yo)
		Q2	Obviously, now you can’t meet up with people and do stuff like that, which is annoying because it would be nice to meet up with people and talk about it a bit more and be a bit normal, sort of thing, but I just try and be as normal as I can, really. ID 3, Week 15, 65yo
**Experience of MBC**	**Living with MBC**	Q3	Sometimes, and I have to watch myself, people will moan about something trivial, and they’re moaning to you and you just think, you haven’t got a clue, love. You just haven’t got a clue. ID 13, Week 14, 54yo
		Q4	I can’t pre-empt the future, I can’t really alter it in any way, other than be sensible and do what experienced consultants suggest I do. And really try, which I know sounds silly, but try not even to think about it. Because if you start thinking about it, you come up with all sorts of horrible scenarios. And whether they’re going to come to fruition or not, I can do nothing about. So, I give it very little thought. ID 5, Week 14, 57yo
		Q5	That’s all about it, it’s the quality of life, and I’m not looking for miracles, I don’t want a miracle. And I don’t want to go on a world cruise, I just want simple things. To be able to see my friends, for her to come and have a cup of tea with me, and to see my grandchildren. ID 6, Week 7/end of study, 56yo
	**Value of being on treatment**	Q6	As long as it shrinks more, I'll keep on doing this and it balances out for me. I don't mind doing this and I don't mind limiting myself as long as it works. It’s just a give and take, balances out kind of. ID 9, Week 20, 42yo
		Q7	Some people would put up with the side effects and they blindly cut off the what’s going to happen inevitable. Whereas I’m straight to the point and know what’s happening the inevitable. So I would then weigh up do I live from now, until whatever’s going to happen at the end without side effects, or do I be where I am now and carry on with a load of side effects. I would definitely not have carried on with side effects with the treatment if they were severe. ID 12, Week 18, 62yo
		Q8	Basically what I’m tolerating is worth keeping on the full dose and giving that the best shot at the moment. ID 7, Week 15, 58yo
		Q9	This is what I was complaining about. With them taking me off and on, it is not giving it a chance to work. I said I am prepared to put up with any of the side effects. I will take allergy tablets. I don’t mind sitting on the loo, I just need it to work. ID 4, Week 22, 62yo

**Table 3:** Supporting quotes for themes of side effects, side effect management, and treatment information and support

Theme	Sub-theme	Ref	Illustrative quote
**Side effects**	**Diarrhoea**	Q10	Well I just think I’ve been very lucky because I’ve never ever had diarrhoea, never since I’ve started on the drugs. ID 18, Week 17, 81yo
		Q11	I’ve had some diarrhoea but it’s not on a regular basis. So, nothing where I’ve had to do any major interventions or affecting the quality of my life, really. ID13, Week 14, 54yo
		Q12	Sometimes by the time I’ve actually got to the toilet I’ve actually started to do it, I’ve got no control it’s just coming. And that’s bad because like I say you don’t want to make a mess of yourself and I definitely wouldn’t want to, it frightens me going outside in case I get caught out because you don’t take spare clothes with you, do you? ID 19, Week 13, 62yo
		Q13	When you’re in pain, I suppose you can go somewhere and be in pain, can’t you? I think with the diarrhoea, that’s very difficult to deal with, because it’s not something that can wait. ID 11, Week 16, 63yo
	**Other side effects**	Q14	It started literally within ten minutes of taking it, the acid was like something else. Yes, like whatever it was definitely trying to eat its way out, it was so bad. I’ve never experienced anything like it. And then my eyes would swell up, the whole of my face would swell up, even under my jaw line going into my neck, it would all swell up, the inside of my mouth. ID 2, Week 13, 60yo
		Q15	What was happening is I was having the tablets when I woke up in the morning. And then my appetite was suppressed. And then I’d have the tablets in the evening before dinner. And my appetite was suppressed. So, in the very beginning, I had a really low appetite. ID 17, Week 11/end of study, 56yo
	**Fatigue**	Q16	And I have just recently started to feel a lot more fatigued. Whether it’s the lack of food, or whether it’s the build-up of the chemotherapy chemicals in my body, I don’t know. But the last ten days or so, I think I have started to think, you know what? I’ve just got out of bed, I slept well all night, and I feel absolutely shattered. ID 5, Week 14, 57yo
		Q17	Sometimes I am struggling, after a few hours, like three or four hours if I work, after that I feel very tired with less energy, and I look like I’m pushing myself. ID 8, Week 13, 48yo
	**Understanding cause of side effect**	Q18	really, I’m quite mardy and low. But that could be just these antidepressants. I don’t think it’s the tablets, really. I think I probably use that as an excuse, if I’m being honest. ID 20, Week 13, 58yo
		Q19	I think initially, when I first went on the Abemaciclib, I did think that I was getting a tired a bit more easily than I used to do, but I don’t know whether that was psychological because that’s what I’d been told. That’s what the drug side effects on the little packets say. Fatigue is a big thing. ID 14, Week 14, 63yo
		Q20	I’m not sure which is causing it, because I started all the treatments at the same time, I don’t know who is the guilty one. But mainly because it can be either. I check the side effects for all of them and it’s any of those can cause the side effects that I have. ID 10, Week 17, 60yo
	**Expectations and perceptions**	Q21	But it’s not as bad as I thought it would be. When I first started and they told me about the tablets, I looked at all the side effects and I thought it was going to be worse, actually. ID 3, Week 15, 65yo
		Q22	I’m actually in the fourth month now of it and I’m finding it surprisingly easier than the oncologist led me to believe at the beginning, which is good. ID 14, Week 14, 63yo
		Q23	I’m lucky because I’ve got friends and they’ve had a horrendous time, I just feel I’ve been lucky from that point of view. ID 18, Week 17, 81yo
		Q24	I would just say the biggest thing has been the massive quality of life impact that this drug has with compared to chemotherapy, and for me it’s a complete and utter game-changer. ID 13, Week 14, 54yo
	**Tolerability of treatment**	Q25	All the literature says that it’s much worse at the beginning, and then the body gets used to it. That has happened to a certain extent, but obviously I’m not free from taking the Loperamide yet, and I don’t know whether I ever will be. ID 7, Week 15, 58yo
		Q26	from May onwards, things started to settle down and at the beginning I used to write down every time I have a tablet, a pill, this and that, how many. Now you just get used to it and it’s just back to general life, really. I don’t even start thinking about it anymore. ID 10, Week 17, 60yo
		Q27	if the diarrhoea was more frequent but manageable, that would be preferable rather than it just be unexpected and an emergency and things happening to me. And if, at any point, I just couldn’t predict it and I would suddenly have to go and change all my clothes and everything that would’ve been something I would’ve really, really struggled with. ID 15, Week 17, 44yo
		Q28	I’ve been on all different chemos since the start of my problem with this cancer and I think this has been the worst for me. When I say the worst, now it isn’t, but the first two months at least it was pretty rough. There was a time when I would have actually said, I’ve had enough, I can’t do this, which isn’t like me. But then now, with the third month and going into the fourth month, yes, it’s much better. I think it’s tolerable now. I still have days that it goes off. I’ll be good for a bit and then I’ll have a bad day, but it’s not like it was before by any sense. Much better. ID 16, Week 13, 76yo
		Q29	It was just that basically I just had to find the right balance, and just go through the cycle with my tablets, with the treatments. I had to just get used to it. ID 1, Week 13, 50yo
**Side effect management**	**Diarrhoea management**	Q30	But when they gave me my first prescription of the Abemaciclib, they did give me Loperamide as well and said, this is available to take should you get the bad diarrhoea. ID 14, Week 14, 63yo
		Q31	I tend to pre-empt things and just take a lot of the Loperamide, not a lot but more than I would do… I think that as long as I am not causing myself other problems by continuing to take the Loperamide, yes, I can tolerate this. If I’m not causing long-term damage, to keep taking the drug to control the diarrhoea, then I will continue to do that. And I have asked. ID 7, Week 15, 58yo
		Q32	I had to make sure I was near a bathroom, like if I was shopping. And made sure I was near a shop, a supermarket, or whatever, near the bathroom. ID 1, Week 13, 50yo
		Q33	I’ve carried on more or less as normal. If I have to go somewhere there’s not a toilet, I have to go somewhere there’s not a toilet. I’ve actually put carrier bags in the boot of my car so that I can use one of those in a dire emergency. ID 2, Week 13, 60yo
	**Dietary changes**	Q34	I don't think that helped when I was not having a good diet. I was eating a lot of chocolate biscuits, and a lot of maybe fatty stuff. But you do need a healthy diet when you're taking them. ID 1, Week 13, 50 yo
		Q35	Sometimes I eat properly, sometimes I can’t eat. I don’t feel like it, especially meat, I can’t eat meat. Especially lamb, red meat. I like vegetarian food. In the beginning, when I started, now I’m okay. ID 8, Week 13, 48yo
		Q36	I found some diet boards and some guidance around abemaciclib even. That's what I've been doing. I've been reading those to see how people are doing on abemaciclib, again, after some tips and tricks about diet and so on […] I've got all the books around all kinds of diets that, say, will cure cancer and will be helpful with MS […] nutritionist three times. I went to a couple of food related workshops. I've tried to learn as much as I could. ID 9, Week 20, 42yo
		Q37	No, I’ve been a bit wilful about it. A lot of people get a cancer diagnosis and they go vegan and various different approaches they take to modifying their diet and really looking after themselves. And I am very greedy and I’m not a vegan, and when I got my diagnosis I went out and bought £35 worth of cheese and a big bottle of wine, and came home and ate it all. So, I’ve not cut meat and dairy out of my diet. ID 15, Week 17, 44yo
	**Management of other side effects**	Q38	the itching skin, I put just some cream and that’s it. And the nausea, it’s under control because they gave me something to control the nausea. ID 10, Week 17, 60yo
	**Fatigue management**	Q39	I know now I am usually all right in the morning. I get up and morning is my best time, and then in the afternoon, I start feeling tired. I do as much as I can in the morning, go out and do whatever. I know I’ll go back after dinner and have a rest then. ID 3, Week 15, 65yo
	**Adherence**	Q40	I know they say take it at the same time every day. So that if I take my first one late in the day, and I haven’t got enough hours left to take the second one before you go into bed, then I may skip the second one. But that has not often happened. ID 12, Week 18, 62yo
**Treatment information and support**	**Treatment information**	Q41	Everything regarding this particular treatment that I’m on now the information was sufficient for me to make a decision on what I was going to be doing going forward, and with the help of the doctors to speak to me. If the doctors didn’t speak to me about it I would probably have not gone ahead with the treatment, so they helped as well with the information that I got on the leaflet that made me decide. ID 12, Week 18, 62yo
		Q42	I wasn’t prepared for the pain. They said that I could have diarrhoea and did give me some tablets for it. But the pain was the worst thing, it was excruciating. ID 11, Week 16, 63yo
		Q43	I would have loved a list, if possible, instead of having that one month having to run back and forth. But I would have loved that. It's not like ‘don't eat this list’, but ‘these may cause…’, would be enough. I think that would be good to have. ID 9, Week 20, 42yo
		Q44	I went through it with the doctor and I was there for about an hour, and we discussed all the side effects, from the possibility of blood clots being the worst sort of thing, and just the general, from an upset tummy, diarrhoea. ID 6, Week 7/end of study, 56yo
		Q45	It was only about the diarrhoea, nothing else, and then I’m curious and I read the full list of side effects. I was kind of prepared. But no, people focus mainly on the diarrhoea thing. The information’s mainly on the diarrhoea side. ID 10, Week 17, 60yo
		Q46	The only slightly disturbing thing is it’s continuous and ongoing, is what’s written on my notes. So, I don’t know how long they expect it to go on for. When they would get to a point where they would think, well, if it were going to do its job, it would have done it. Because it would be nice to feel there was an end date on it. But there doesn’t seem to be at the moment. ID 5, Week 14, 57yo
	**Support from HCPs**	Q47	My cancer nurse says, that even when I go back to work, she’s going to write to the school and say, [name] needs Wednesday afternoon off to go to this. Because it’s good for mind, body and soul. That actually made me realise that they are on my side. I’m not just a number. ID 20, Week 13, 58yo
		Q48	She said everyone that I’ve treated is on the Palbo. So, she said, I’ve no experience of it because you’re the only one that I know that’s on it. It was weird, and I’m thinking, well I’ve got no one to turn to here. I’ve got no one to ask. If the nurses don’t know, who have I got to turn to if I’ve got odd symptoms? ID 2, Week 13, 60yo
	**Advice for others**	Q49	I think the thing is to explain to people, don’t be frightened. All drugs and drug therapy, they do sound frightening, especially when they’re on paper. And you’re handed the negativity from it, you’re handed the side effects and things like that. They don’t hand you a piece of paper that says patient X, say me, did it, trialled this drug for so long and she found that it didn’t incur on her life. ID 6, Week 7/end of study, 56yo
		Q50	I would probably say that the best thing to do is go in with a very, very open mind and don’t anticipate. ID 14, Week 14, 63yo

**Table 4:** Supporting quotes for themes of relationship impacts and impact on daily life

Theme	Sub-theme	Ref	Illustrative quote
**Relationship impacts**	**Impact to partner**	Q51	My husband’s doing everything and I do feel sorry for him because it’s not fair. He does everything. And like I say, when I’ve had really bad diarrhoea and it’s gone everywhere, he’s actually physically had to help me, you know to get in the shower, or to clean me up…. we’ve only been married five years, he didn’t sign up to be a carer, he signed up to be my husband. ID 19, Week 13, 62yo
		Q52	last Sunday we were in bed, watching TV, and I passed wind. I had pants on, and it followed through, and into the bedsheets. I was just so embarrassed. He just went, don’t worry about it. But I just, you know, I was just so embarrassed because it’s not. So then, who wants to make love when you have an accident like that. Not me, for a start. So, yes, it did have an impact on it. ID 20, Week 13, 58yo
		Q53	I don’t really like being hugged that much because it hurts my stomach. I have actually said you have got to be careful. It is not only that. If there is too much activity, especially during the evening, and if I am loose, I could poo all over him. We actually joked about it in the beginning when we were going through the side effects when I was starting it. ID 4, Week 22, 62yo
	**Impact to family life**	Q54	Well I really miss them. I mean sometimes like I say you just want a hug or you to give them a hug, because like with my condition I don’t know how long I’m going to be here because they’ll never cure it. It’s like a death sentence so I want to spend as much time as a I can seeing the grandchildren and making memories. And like I say you can’t do that. It’s really hard. ID 19, Week 13, 62yo
		Q55	Sometimes my daughter, she wants to share with me, she’s working, and before she shared everything with me and I was very happy to listen. To tell her, don’t do this, this is good for you. Now when she’s sharing with me, my interest is lost, actually, I don’t want to listen to her. I find it irritating when she’s talking. ID 8, Week 13, 48yo
		Q56	I’ll tell them, oh, yes, I’m doing okay. Even if I’m having a bad day, because there’s no need to worry them. For what reason? It’s not going to help them and it’s not going to help me. So, yes, I try to play up light on it like it’s, yes, I’m doing great. I’m getting out. I’m doing this and doing that. I make it sound like I’m doing more than I am sometimes but, like I say, the distance does help in that they don’t see you when you’re having your rough days. Because they would worry. ID 16, Week 13, 76yo
		Q57	My mum’s 80. You want to protect children, but then there comes a time in your life when you want to protect your parents in the same way. Your relationship changes. When you talk about relationships and you want to be protective, I don’t tell my mum half the things that go on, because I don’t think she needs to know. In her 80 s, she doesn’t need to know, and I don’t want her worrying about it. At the end of the day, there’s nothing worse, as a mother, as seeing your child hurt, ill, or in pain, and we would all want to protect our children from that. ID 6, Week 7/end of study, 56yo
**Impact to daily life**	**Impact to daily life and responsibilities**	Q58	One day the fat lady’s going to sing and there’ll be nothing I can do about that, but until she starts singing I’m going to carry on doing whatever I can, regardless of what the treatment puts in my way. I will find a way round it. Because, well that’s what you have to do. You’ve got to live. You’ve got to live with cancer and you have to live with the treatment that you’re on, and you have to live with the side-effects, otherwise you may as well just go to bed and stay there, and that’s not me. ID 2, Week 13, 60yo
		Q59	Now everything is okay, but in the beginning if my husband asked me, come on, let’s go, he knew I was depressed, he’d say let’s go to lunch or dinner or go out in the park or something, I always refused. I’d say I don’t want to go. I was just scared because of, if that happened. ID 8, Week 13, 48yo
		Q60	But as I say, it just does affect what I can do. Even my housework, I do a little bit and I get more tired and I’ll sit down, and I used to be painting the house top to bottom, doing things like that, and now I think, oh, no, I haven’t got the energy to do that. ID 3, Week 15, 65yo
		Q61	There is no way on earth I can just get up, have breakfast and go out. I have to allow at least two to three hours for my stomach to stop wanting to go to the toilet. Of course, it doesn’t matter when I am at home. ID 4, Week 22, 62yo
	**Impact to social life**	Q62	Yes, I have had less social activity because we used to go visit people and now I’m scared. They have obviously got facilities, but my main concern with that is sometimes when it does go, sometimes it’s okay, other times it’s just like a blast and it just makes a mess everywhere and it’s awful. I always think I don’t want to do that if I’m at somebody else’s home, so I back off going. I have been a couple of times to visit, but maybe just for an hour or two. I don’t stay for long periods at these places. ID 16, Week 13, 76yo
		Q63	It sadly makes you think, when somebody says, shall we go and have some lunch? You think, well, no, actually we won’t, we’ll maybe have a coffee and go home. But that’s it, sort of thing. So, sadly it does affect my life like that. And I haven’t yet been brave enough to go out and eat an evening meal anywhere. ID 5, Week 14, 57yo
		Q64	I didn’t say to anybody I’m not coming out this evening, or I’m not going to a restaurant because I might have diarrhoea. I’d just be a bit more forward planning and make sure that I’ve always had my tablets in my bag as well. But, no, I’ve not let anything get in the way of my life. ID 17, Week 11/end of study, 56yo
		Q65	I’m full of wee streaks, but I just do a bit and then I’ve got to sit down. And I had a stable full of horses at one time I had a very full social life, I must admit I’ve had to curtail that because I just get so tired at night. ID 18, Week 17, 81yo

**Table 5:** Supporting quotes for theme of finances and employment

Theme	Sub-theme	Ref	Illustrative quote
**Finances and employment**	**Employment impact**	Q66	As soon as I got the diagnosis I felt it was them that caused me to stop work more than me, because I wanted to carry on working. I told them the diagnosis, it was more of a shock to them than it was to me I think. They said they couldn’t understand why I wanted to carry on working, and I went well I suppose it takes your mind off everything. And I wanted to but I felt they talked me out of working more than me, I would have carried on. So I stopped and that was it, that’s where we are now. ID 12, Week 18, 62yo
		Q67	I didn’t tell any people and definitely didn’t tell at work because I would not get any work. ID 7, Week 15, 58yo
		Q68	But hopefully I'm going to go back to work, because I think maybe I've been all right on it. I'm hoping that I'll be fine, the side effects will be all right, and it won't be so bad. And I can go to work. ID 1, Week 13, 50yo
	**Financial impact**	Q69	But Macmillan called me and told me that I was entitled to some money every week or something. I think it’s 80-something quid anyway. I said, no, we don’t need it. But she said, no, you’re entitled to it. So it does help, I must admit, especially with the costs going up like it is. It will definitely be probably something I won’t be sorry to get. But we can get by. ID 16, Week 13, 76yo
		Q70	I am panicking like mad deep down. Am I going to get any benefit or not? I did actually get a text message three weeks ago from PIP to say they have received my form, but nothing has happened. All I get is ‘well, it takes about six weeks for it to go through’. I am constantly checking my wages, am I coping at the minute? Can we cope? It is a good job I am only eating small food. I am only buying what I need to buy and what is essential at the minute. I don’t go anywhere which helps, doesn’t it? We don’t have holidays and he doesn’t either. We are watching pennies carefully at the minute. I don’t smoke, obviously and I don’t even have alcohol now. No treats. ID 4, Week 22, 62yo
		Q71	I have to rely on other people to take me, and I have to pay their petrol, or their day off work. Which then does take out of my low-paid job. But you know, and we’re very lucky we don’t pay for our medication. Because I’d hate to think what they would cost if we lived in America. ID 20, Week 13, 58yo

Themes of MBC and COVID- 19 influenced the context of what participants reported. We coded these separately and present them first, acknowledging the backdrop they provide to participant interviews.

### COVID- 19

Obviously, now you can’t meet up with people and do stuff like that, which is annoying because it would be nice to meet up with people and talk about it a bit more and be a bit normal, sort of thing, but I just try and be as normal as I can, really. ID 3, Week 15, 65yo

Interviews were conducted between 2020 and 2023 so the pandemic was mentioned by over half of participants; in particular, there were discussions about the limitations this imposed on both clinical and social interactions (Table 2: Q1, Q2). Having to avoid people who might have been unwell weighed heavily knowing that time with family and friends might be limited. The added worry also materialised in additional cleaning practices and precautions.

### Experience of MBC

Sometimes, and I have to watch myself, people will moan about something trivial, and they’re moaning to you and you just think, you haven’t got a clue, love. You just haven’t got a clue. ID 13, Week 14, 54yo

Heightened anxiety about COVID added to that of living with MBC. Participants were mindful of their vulnerability, avoiding activities which may pose danger. However, despite feeling worried, people spoke about appearing ‘normal’ which meant those around them did not acknowledge how difficult living with MBC could be (Table 2: Q3).

There were also references to uncertainty about the disease trajectory (Table 2: Q4). This highlighted certain priorities for people, particularly remaining on treatment (Table 2: Q5). Ambiguity about the future fed into a commitment to abemaciclib, expressing belief about its efficacy (Table 2: Q6). Some perceived treatment as preventing death, whereas others would not take any drugs which might compromise their QoL (Table 2: Q7). Participants considered a point at which side effects would be too burdensome or a stoicism to endure whatever happened to remain on treatment (Table 2: Q8). Those discontinuing felt remorse that it had not worked and those needing treatment breaks were frustrated the drug had not been given ‘its best shot’ (Table 2: Q9).

### Side effects

Sometimes by the time I’ve actually got to the toilet I’ve actually started to do it, I’ve got no control it’s just coming. ID 19, Week 13, 62yo

The most prominent side effect discussed was diarrhoea, but experiences were mixed with some participants reporting none (Table 3: Q10) and others that it was either manageable (Table 3: Q11) or debilitating (Table 3: Q12). Diarrhoea was often accompanied by pain, nausea, and shame about potential public embarrassment (Table 3: Q13). The latter was based on perceptions; uncontrollable and unpredictable episodes were not always realised and limited to a few participants.

What was happening is I was having the tablets when I woke up in the morning. And then my appetite was suppressed. And then I’d have the tablets in the evening before dinner. And my appetite was suppressed. So, in the very beginning, I had a really low appetite. ID 17, Week 11/end of study, 56yo

Other gastric side effects were highlighted—heartburn, nausea, and cramping, which were intense and disruptive (Table 3: Q14). There was discussion of weight and appetite changes, with surprise at weight gain alongside diarrhoea (Table 3: Q15).

Some participants had fatigue to an extent where rest was not restorative (Table 3: Q16). This stemmed from disrupted sleep or a pervasive lack of energy and was frustrating for those who felt unable to do things (Table 3: Q17).

A persistent narrative saw participants grappling to understand the cause of their side effects. There was a consensus ascribing diarrhoea to abemaciclib, with some question of dietary influence. Attributing the cause of other side effects was more difficult (Table 3: Q18). For example, participants thought fatigue could be due to treatment, the emotional valence of MBC, age, and/or lifestyle (Table 3: Q19). Often taking multiple treatments, participants struggled to discern which side effect was caused by which medication, particularly with overlapping toxicity profiles (Table 3: Q20).

I’m lucky because I’ve got friends and they’ve had a horrendous time, I just feel I’ve been lucky from that point of view. ID 18, Week 17, 81yo

For some, side effects were not as bad as initially expected so treatment was largely perceived as better than anticipated (Table 3: Q21, Q22). Comparison was often made to other people (Table 3: Q23) or chemotherapy perceived as a worse option (Table 3: Q24). Participants described a shift in side effects, improving with time (Table 3: Q25, Q26). Consideration was given to the point at which side effects become untenable (Table 3: Q27); emphasis was on tolerating discomfort until side effects either improved or stabilized (Table 3: Q28, Q29).

### Side effect management

I’ve carried on more or less as normal. If I have to go somewhere there’s not a toilet, [then] I have to go somewhere there’s not a toilet. I’ve actually put carrier bags in the boot of my car so that I can use one of those in a dire emergency. ID 2, Week 13, 60yo

Participants cited loperamide as the key management strategy for diarrhoea (Table 3: Q30). Several people took this prophylactically though one worried about it causing harm (Table 3: Q31). People took other precautions when going out, such as waiting to leave the house until after they had emptied their bowels, staying close to places with toilets (Table 3: Q32), or carrying personal hygiene cleaning supplies with them (Table 3: Q33).

Dietary alterations were made to what, when, and how much food was eaten (Table 3: Q34). Some changes resulted from altered taste, diminished appetite, and mouth ulcers. There was variability within and between participants as to which foods they felt triggered diarrhoea (Table 3: Q35). A few participants monitored this closely (Table 3: Q36), while others took a more relaxed approach (Table 3: Q37).

Skin rashes and mouth ulcers were remedied by creams and mouthwashes (Table 3: Q38). Allergic reactions were controlled with antihistamines and steroids. Rest and staggering events were keys to managing fatigue (Table 3: Q39). Some side effects, like hot flushes, seemed harder to alleviate.

Largely participants adhered to abemaciclib, though there were dose reductions and treatment breaks as directed by treating clinicians. Some implemented strategies to ensure they took both tablets a day routinely, whereas others were more pragmatic, believing that a missed or delayed dose would be acceptable (Table 3: Q40).

### Treatment information and support

I went through it with the doctor and I was there for about an hour, and we discussed all the side effects, from the possibility of blood clots being the worst sort of thing, and just the general, from an upset tummy, diarrhoea. ID 6, Week 7/end of study, 56yo

Participants spoke of being informed about the possibility of diarrhoea (Table 3: Q41), though the intensity of it was sometimes misunderstood (Table 3: Q42). There was variation in how much information people were given or wanted (Table 3: Q43, Q44). Some of the rarer side effects, like allergic reactions and blurred vision, were absent from this (Table 3: Q45). There were other vagaries surrounding treatment, particularly when to expect it to start or stop working (Table 3: Q46).

Part of this ambiguity stemmed from clinical support available. While some participants felt their needs were well managed (Table 3: Q47), others had to chase for information. There was a sense of vulnerability if healthcare professionals, including general practitioners, lacked experience with abemaciclib (Table 3: Q48). A few participants did not seek support, managing things by themselves.

Participants offering advice to others suggested maintaining an open mind, rather than being fearful about possible side effects (Table 3: Q49, Q50). A sense of perseverance was apparent, knowing things may improve.

### Relationship impacts

Last Sunday we were in bed, watching TV, and I passed wind. I had pants on, and it followed through, and into the bedsheets. I was just so embarrassed … who wants to make love when you have an accident like that. Not me, for a start. ID 20, Week 13, 58yo

Support from partners, especially those with whom participants had intimate relationships, was frequently mentioned. Partners were often required to undertake new responsibilities, largely in relation to practical tasks but extending to more personal support, such as assistance following an episode of diarrhoea (Table 4: Q51). There was a clear impact on the sex lives of participants, largely due to embarrassment around diarrhoea, be it cleanliness or unpredictability (Table 4: Q52). Others discussed soreness, making intimacy difficult, if not impossible. This extended to reduced physical touch, such as hugging and kissing (Table 4: Q53). There was guilt expressed around this. The quotes referenced here refer to discomfort from diarrhoea, but participants also noted vaginal discomfort associated with accompanying hormone therapy.

I’ll tell them, oh, yes, I’m doing okay. Even if I’m having a bad day, because there’s no need to worry them. For what reason? It’s not going to help them and it’s not going to help me…Because they would worry. ID 16, Week 13, 76yo

There was a wider discussion of effects on family dynamics, be it a desire to be closer to (Table 4: Q54) or feeling frustrated by relatives (Table 4: Q55). Some participants screened information disclosed to family, either withholding details or tempering the severity, often dependent on the generational differences present (Table 4: Q56, Q57). There was an assessment of what information to share with others and who it would ultimately benefit. For example, one participant refrained from imparting information to an elderly parent while another held back all disclosure from their family entirely.

### Impact on daily life

Now everything is okay, but in the beginning if my husband asked me, come on, let’s go, he knew I was depressed, he’d say let’s go to lunch or dinner or go out in the park or something, I always refused. I’d say I don’t want to go. I was just scared because of, if that happened. ID 8, Week 13, 48yo

Participants were split into either maintaining life as normal or feeling limited (Table 4: Q58, Q59). Largely, participants valued independence, only accepting help where necessary (Table 4: Q60). This was reflected by those continuing to go out and use public bathrooms, compared to those who felt hesitant (Table 4: Q61). There was embarrassment related to diarrhoea in social settings, be it about an accident happening, needing to clean themselves, or feeling the need to vomit (Table 4: Q62). These apprehensions were either based on fears about its occurrence or actual experience.

Adaptations were made to remain social, such as avoiding meeting at certain times of day, keeping visits limited to the home, or not meeting for a meal (Table 4: Q63, Q64). Some individuals had less energy to meet people while others noted the absence of anyone to talk to (Table 4: Q65).

### Finances and employment

I didn’t tell any people and definitely didn’t tell at work because I would not get any work. ID 7, Week 15, 58yo

Those who were employed either stopped working upon diagnosis or continued throughout, particularly if working from home (Table 5: Q66, Q67). Those on sick leave contemplating a return to work assessed the practicalities of this, such as the availability and accessibility of toilets (Table 5: Q68).

Participants spoke about financial security if they had retired early or had other support in place (Table 5: Q69). However, some felt an extra burden, particularly if they needed to navigate the benefit system (Table 5: Q70). In considering their own difficulties, people drew comparisons to others, be it those who were perceived to have financial plans in place or those who would find it more difficult. For example, participants noted the benefit of not needing to pay for medication, contrasting this to those in the USA (Table 5: Q71).

## Discussion

Other research into CDK4/6 inhibitors has predominantly focused on palbociclib whereas this analysis addressed the gap of understanding what treatment is like with abemaciclib. Work which has focused on abemaciclib has stemmed from clinical trials, emphasizing diarrhoea management. As the first real-world study into this treatment, we are able to provide novel insight into the ways people coped and tolerated the drug while understanding the value they placed on this. There was a heavy emphasis on the perceived importance of treatment and what people were therefore willing to tolerate to ensure they could stay on it. The way people conceptualise their treatment informs a great deal of how they engage with it [25, 26].

Previous research into the various CDK4/6 inhibitors used in the MBC setting has demonstrated better adherence than expected [12, 15]. There are correlates linking this to both treatment belief and successful integration into daily routine [15]. Qualitative research with patients in the USA living with MBC, and taking a CDK4/6 inhibitor, showed that participants discussed adherence to treatment in the context of the importance of staying on their medication [15, 16]. However, the majority of the samples included have been treated with palbociclib. The quantitative data from our study showed that those who remained on abemaciclib were willing to take steps to ameliorate side effects with few occurrences of repeated poor adherence [20]. Our qualitative analysis provides nuanced detail about why people did this this and the mental processes people used to remain on treatment. Up until now, this understanding has been missing in the literature about this treatment.

Theories about illness beliefs and adherence, such as the common-sense or self-regulation model, highlight both emotional and cognitive components [25–27]. As previously shown, a belief in the necessity of a treatment can outweigh the impact of side effects [12, 28, 29]. This was reflected in our interviews by participants who spoke about their coping and management techniques, be it emotional strategies to engage with the challenge of treatment through reframing or practical ones such as implementing strategies to follow the correct dosing schedule for abemaciclib, demonstrating an element of control [30, 31]. Participants did acknowledge times they had missed or forgotten tablets, mirroring other research into adherence [12, 32]. However, it was evident that participants valued being on treatment, conceptualising it as a lifeline and placing a strong belief in its efficacy. This position of hope has been considered a mitigating factor against poor adherence and treatment discontinuation [33, 34].

Understanding the emphasis put on treatment can also explain management of side effects [2, 29]. Patients perform their own risk/benefit analysis and the advanced nature of the disease may lead to a prioritisation of remaining on treatment in spite of side effects [28]. For those living with MBC, extending survival may be the most important goal [35]. IMPACTOR participants mirrored this, referencing their willingness to persevere with side effects to remain on treatment. It is also important to note that having good QoL is what makes progression-free periods meaningful for people [35]. We saw assessments of this when participants described a point at which side effects would have been too overwhelming, referencing hypothetical scenarios deemed as unmanageable, yet these were always a step beyond their experiences. This may be unique to our cohort, as outlined in the limitations. However, reframing strategies like this have been linked to psychological resilience and improved quality of life within a cancer setting [34, 36]. This moves to viewing treatment as manageable was evident in the various strategies people took to incorporate the drug into their daily life, such as avoiding going out at certain times of day to avoid diarrhoea in public.

Diarrhoea has been highlighted as the most prominent side effects in clinical trials of abemaciclib [5, 6]. Our qualitative findings show that participants seemed to anticipate this and felt informed. Fatigue was also referenced throughout and again, seemed to be accepted. Other side effects however, such as allergic reactions, were more surprising and our analysis exposed informational gaps. Psychoeducation is a key component to patient outcomes and wellbeing [31]. Participants in this study talked about the importance of the information provided to them in knowing how to manage side effects. Treatment was harder to tolerate when the cause or expectations of a side effect were ambiguous. There was variability in how much information was shared and knowledge of where to access this. In the metastatic setting, there is often a need for additional information and opportunities for support [3]. For example, there was a notable lack of discussion about the aforementioned impact on intimacy, a recurring omission in the metastatic setting [37].

As part of persevering with abemaciclib, relationships with partners and the wider family group were referenced. There appeared to be more open dialogue and shared experience with partners, perhaps reflecting a move to dyadic coping with both individuals able to engage and express [38]. Some spoke about their gratitude in the support available within their relationship, primarily in reflection of a shift in responsibilities. Others felt they were the ‘strong one’ in the partnership and therefore were not offered emotional support. Wider family communication was a balance of the type and amount of information people needed to know, such as sharing less information with children to protect them.

Emotional support from friends was a surprisingly limited topic of conversation. Participants did speak about maintaining social connections, be it altering the duration or type of meeting. For some, being able to get out was a welcome distraction, whereas for others, there was increased frustration. There was an element here of navigating these meetings in light of the COVID- 19 pandemic however, so some sense of inherent limitation.

## Limitations

It is important to note the material discussed here was from a small sample of those participants opting in to an interview. There may have been an element of demand characteristics in speaking about adherence [39, 40]. As our group was self-selecting, those who missed or skipped doses may not have wished to speak to us and there is the possibility of introducing bias [15]. What remains unknown is how those people who did not take part, in either the study or the interview, coped with treatment and whether there were intrinsic differences in the groups. There is a suggestion that those who are more concerned about treatment burden are less likely to adhere and remain on treatment; however, those in a metastatic setting have likely been exposed to more lines of treatment and therefore are more knowledgeable about expectations [2]. Other qualitative work suggests that sociodemographics may also underline these differences, something which should be considered within our sample which is largely homogenous [15].

## Conclusion

Our interview findings provide the first real-world insight into how patients living with MBC manage treatment with abemaciclib, alongside ET. While other studies have focused on toxicity and survival data, these interviews illuminate what it is like to be on treatment. What was salient throughout was the belief in abemaciclib and the importance placed on being on treatment. Coping with treatment was perceived as more difficult when information or support was required from other people. Specifically, participants felt isolated if medical professionals were inexperienced with the drug or if their main focus was on diarrhoea. Unlike more prominent treatments, there were fewer opportunities for peer support with others taking abemaciclib. Future work could be undertaken to develop peer-led resources to facilitate this.

This work can inform development of future support for patients. In particular, the ways participants described managing treatment and side effects reflect the duality of coping, making both practical and emotional adaptations. Further exploration of this theoretical underpinning could allow for targeted support, particularly in considering all the roles and facets of life which may be impacted in the real-world application of treatment. As other research suggests, supporting patients living with metastatic breast cancer requires a multi-dimensional approach [14].

## Supplementary Information

Below is the link to the electronic supplementary material.

Supplementary file1 (JPG 76 KB)

Supplementary file2 (DOCX 22 KB)

## Data Availability

The datasets generated during this qualitative study are not publicly available to protect anonymity. Participants did not consent for data to be shared or used for future research.
