# Supplementary figures and images for: Exploring the real-world experience of abemaciclib treatment for HR +, HER2 − metastatic breast cancer—a qualitative analysis of the IMPACTOR study
Source: Support Care Cancer. 2025 Apr 26;33(5):421. doi: 10.1007/s00520-025-09444-3 (PMC12033103; doi:10.1007/s00520-025-09444-3)

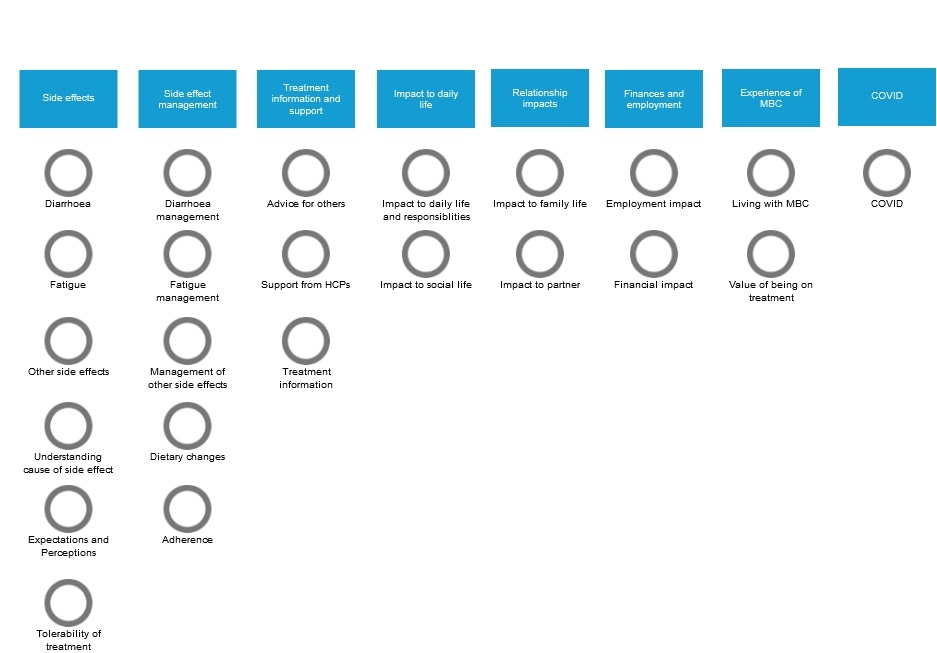

Supplement: Supplementary file 1 — Supplementary file1 (JPG 76 KB) [file 520_2025_9444_MOESM1_ESM.jpg]
