# Supplementary material for: Exploring the real-world experience of abemaciclib treatment for HR +, HER2 − metastatic breast cancer—a qualitative analysis of the IMPACTOR study
Source: Support Care Cancer. 2025 Apr 26;33(5):421. doi: 10.1007/s00520-025-09444-3 (PMC12033103; doi:10.1007/s00520-025-09444-3)
Supplement: Supplementary file 2 — Supplementary file2 (DOCX 22 KB) [file 520_2025_9444_MOESM2_ESM.docx]

# S1 - IMPACTOR Interview Topic Guide

*At this point, researcher and participant have already discussed the study and any questions arising and participant has given signed informed consent.*

**Main body of interview:**

The interview will begin with an open question, inviting participants to tell the story of their experiences of the new treatment regimen.

Follow on questions will then arise directly from the participants’ narrative (internal questions). External questions, on topics of interest to the research but not raised by the participant during their initial narrative, will also be included.

External questions will be organised with open questions around eight top level discussion topics. In each case the participant will be invited to discuss the topic freely. Top level discussion topics are listed below, along with some probes that the research may use to further explore each topic, if the participant doesn’t naturally touch on them. The interviews, conducted by experienced qualitative researchers, will be responsive to the participant’s narrative so will not follow a fixed format.

Examples in spoken English are just that, each interviewer should adapt the guide to fit their own style and add in probes to explore topics further based on the conversation with the participant.

**Experience of side-effects and strategies to manage them** (e.g. in spoken English: did you experience any side-effects that you felt might have been from the treatment? What did you do to cope with them?)

Coping strategies employed (including non-adherence)

If participant discussed non-adherence, explore this in the context of beliefs about the need for and efficacy of treatment

Whether participant attributes side effects solely to abemaciclib, or to ET, or to both

Role of healthcare profession in managing side effects

Any support you would have liked to manage your experience of treatment

What information were you given about potential side effects?

How well did you feel the information prepared you for the side effects you experienced?

What, for you, would be a meaningful improvement in this side effect? What is manageable and tolerable for you?

**Impact of treatment on everyday roles and responsibilities** (e.g. in spoken English: did you feel that the treatment affected how you were able to go about everyday activities, such as doing the shopping, getting out and about? How did you manage/feel about this?

**Impact of treatment on family life and family role function** (e.g. in spoken English: did you feel the treatment affected your family life and what you routinely do in the family? In what way?

Ability to provide usual care and support to others

Concerns around the emotional impact for family members

**Impact of treatment on personal relationships** (e.g. in spoken English: do you think that your personal relationships (e.g. with your partner) have been affected since you started treatment? On an emotional level? On a physical/sexual level)

**NTI:** this topic may not be appropriate for discussion with all participants and should be approached with sensitivity.

**Impact of treatment on ability to manage financial commitments** (e.g. in spoken English: did the treatment interfere with your ability to manage the family/your finances? Do you have worries about your/your family’s financial future?

Worries about financial future

Impact of financial concerns on family

**Impact of treatment on working responsibilities and career (where relevant)** (e.g. in spoken English: how has your job been affected? How do you feel about your future career/job opportunities?

Feeling able to do your job as well as you would like

Opportunity costs such as missed promotions, not applying for new jobs

**Impact of treatment on social life and sense of quality of life** (e.g. in spoken English: what impact has treatment had on your social life and overall quality of life? How?

Ability to maintain social and leisure activities? What about getting around – public transport etc.

Impact on emotional health and wellbeing

Of all the impacts you’ve discussed, which one bothered you most and why?

**Expectation vs. experience of treatment** (e.g. in spoken English: looking back, were your expectations from the treatment the same as what you actually experienced? In what way did they differ?)

Worthwhile nature of treatment in the context of side effects experienced

Change in expectations and experience since beginning treatment

Health beliefs – in particular beliefs around the need for and efficacy of treatment and concerns around medication

Role of health care professionals in forming expectations/beliefs about medication – e.g. communication and information giving

**Closing the interview:**

Are there any important issues that we haven’t covered or that you would like to expand upon?

Is there anything else you would like to say that you think is important for the research?
